# Incidence of Thromboembolism in COVID-19 Patients in Intensive Care Units: A Retrospective Cohort Analysis

**DOI:** 10.7759/cureus.47014

**Published:** 2023-10-14

**Authors:** Ayse Z Turan Civraz, Ipek Duzyol, Emine Atli, Cigdem Caglayan, Emine Ozer Yurt, Adnan Ata, Mehmet Yilmaz, Berna Karakoyun

**Affiliations:** 1 Department of Anesthesiology and Reanimation, Kocaeli City Hospital, Kocaeli, TUR; 2 Department of Physiology, University of Health Sciences, Hamidiye Faculty of Medicine, Istanbul, TUR

**Keywords:** coronavirus disease 2019 (covid-19), venous thromboembolism (vte), arterial thromboembolism, variants of covid-19, covid-19 induced thromboembolism, covid-19 associated coagulopathy

## Abstract

Introduction: Coronavirus disease 2019 (COVID-19) infection was declared a pandemic, causing high mortality and morbidity worldwide. It predisposes patients to both arterial and venous thromboembolism, which causes high mortality, and is one of the most serious complications of the disease.

Objective: The aim of this retrospective study was to determine the frequency of thromboembolic events in patients diagnosed with COVID-19 in the intensive care unit (ICU) and to identify the factors causing thromboembolism.

Material and methods: The digital records of patients admitted to the adult ICU of Derince Training and Research Hospital, Kocaeli, Turkey, with a diagnosis of COVID-19 between March 13, 2020, and December 31, 2021, were retrospectively reviewed.

Results: Data of 484 patients, 248 (51.2%) female and 236 (48.8%) male, aged between 18-98 years were analyzed. The overall, arterial and venous incidence of thromboembolism was 14.8%, 11.3%, and 3.5%, respectively. There was no significant association between COVID-19 variants and the development of thromboembolism. The effect of various patient variables on the development of thromboembolism was evaluated, including cardiovascular disease (p<0.001), age (p=0.003), use of acetylsalicylic acid (ASA) (p<0.001), antiplatelet therapy (p<0. 001), acute physiology and chronic health evaluation (APACHE) II score (p=0.003), D-dimer (p=0.015), fibrinogen (p=0.032), ferritin (p=0.015), prothrombin time (PT) (p=0.015), international normalized ratio (INR) (p=0.012), troponin (p=0.012) values at the ICU admission were found statistically significant. The cut-off values were 2.565 (μg/mL) for D-dimer, 435.51 (mg) for fibrinogen, 633.55 (ml/ng) for ferritin, 1.155 for INR, and 0.085 (ng/mL) for troponin.

Conclusion: Although low-molecular-weight heparin (LMWH) is the first choice, it may be appropriate to add ASA and other antiplatelet agents to reduce the risk of thromboembolism in patients with high thromboembolic risk including advanced age, cardiovascular disease, and elevated levels of D-dimer, troponin, ferritin, and fibrinogen.

## Introduction

Coronaviruses are enveloped RNA viruses. They are known to cause disease in animals. In humans, some types have been identified that primarily cause mild and endemic respiratory infections [1,2]. However, severe acute respiratory syndrome-associated coronavirus (SARS-CoV) in 2002-2003 and Middle East respiratory syndrome-related coronavirus (MERS-CoV) in 2012 caused high mortality that progressed to acute respiratory distress syndrome (ARDS) and death [3]. In 2019, another coronavirus variant was detected in the Wuhan province of China, causing a pandemic [4]. The virus, which primarily causes severe pneumonia, had a high mortality rate worldwide. According to the World Health Organisation (WHO), 769 million cases were detected worldwide and 6.9 million people died as of August 2023 [5]. Clinical symptoms of the disease can be mild, such as sneezing, coughing, and fever, or severe, such as pneumonia, ARDS, multiple organ failure, and death [6].

As the disease spread worldwide and became better understood, reports were published that coronavirus disease 2019 (COVID-19) infection can predispose to thromboembolism and cause widespread microthrombi in the body [7]. Patients with coagulopathy have been reported to have a more severe pneumonia and a worse prognosis. Patients admitted to intensive care units (ICUs) also had worse coagulation parameters than those not admitted to ICUs [8]. Arterial and venous thromboembolism were also reported to be more common in COVID-19 patients This is mainly attributed to endothelialitis and cytokine storm due to immune response [9].

Although there are many studies on COVID-19 and thromboembolism, there are no studies investigating the effect of COVID-19 variants on thromboembolism. The primary objective of this study was to investigate the effect of different COVID-19 variants on the frequency of thromboembolic events in patients followed up in ICUs. In addition, we wanted to determine the overall frequency of thromboembolic events in patients diagnosed with COVID-19 in our ICUs and whether different doses of antiaggregant/antiplatelet/anticoagulant therapies and patients' comorbidities had an effect on the frequency of thromboembolic events.

This study was presented as an oral presentation at the First International Medicine and Pharmacy Congress, 2023, Istanbul, Turkiye.

## Materials and methods

Study design and participants

This retrospective study is an observational analytical study which was designed as a retrospective cohort study. After approval by the Kocaeli Derince Training and Research Hospital Ethical Committee of Clinical Research (KDEAH-KAEK 2022/5), patients older than 18 years who were admitted to the ICUs of Derince Training and Research Hospital, Kocaeli, Türkiye, with a diagnosis of COVID-19 between March 13 2020, the date of admission of the first patient with a diagnosis of COVID-19, and December 31, 2021, the date of the last patient's admission before applying for a study permission, were included in the study. The inclusion criteria were admission to the ICU with a diagnosis of COVID-19 between the specified dates and being over 18 years of age. Patients under 18 years of age were excluded.

Data collection and definition

Since identifying information was not included in this retrospective study, informed consent was not obtained from the patients. Instead, separate permission was obtained from the hospital directorate to use the hospital archive and from the Ministry of Health of the Republic of Turkey for the use of patient records to conduct a study on patients diagnosed with COVID-19. First, the list of patients admitted to the ICU with a diagnosis of COVID-19 in the specified time period was requested from the data processing centre of our hospital, and then the necessary data were collected by the researchers from the digital archive of the hospital. Patient demographics, including name/age/gender, comorbidities, and date of diagnosis and ICU admission, were collected to answer our research question of how the COVID-19 diagnosis was made and which COVID-19 variant, if any, was sought. Before analysis, the data of the patients were de-identified and the patients were numbered.

To identify additional comorbidities, the diagnoses found in the patients' medical records prior to admission to the ICU were used. Patients' comorbidities were categorised: (i) Cardiovascular system (CVS) diseases, which included congestive heart failure, coronary artery disease, and arrhythmias; (ii) Neurological diseases, which included all types of dementia, previous cerebrovascular events, and neurodegenerative diseases; (iii) Respiratory system diseases, which included asthma and chronic obstructive pulmonary disease (COPD); (iv) Endocrine and rheumatological diseases, which included hypo/hyperthyroidism and rheumatological diseases; and (v) Metabolic diseases, which included obesity. Hypertension, diabetes mellitus (DM), pregnancy, malignancy, and renal diseases were added as separate categories.

In our hospital, the diagnosis of COVID-19 was made according to the WHO guidelines, which divided patients into three groups: suspected diagnosis, probable diagnosis, and confirmed diagnosis [10]. During data collection, the diagnosis classification of patients was also subregistered in accordance with the guidelines.

Anticoagulant/antiplatelet therapy received by the patient was recorded. The first choice anticoagulant was low-molecular-weight heparin (LMWH) and the second choice was heparin. The treatment regimen was applied in two ways: prophylactic and treatment dose, depending on the patient's clinic. For prophylactic treatment, the dose was given as enoxaparin 40 mg 1x1 subcutaneous (sc.) if the patient's BMI was <40kg/m^2^, and enoxaparin 40 mg 2x1 sc. if the patient's BMI was >40kg/m^2^. The treatment dose was increased by 25-30% above the prophylactic dose in patients with thromboembolic complications, patients at high risk of developing thromboembolic complications, or patients with recurrent catheter occlusion despite prophylactic treatment. Patients with current bleeding or platelet counts <25-30,000/µl did not receive anticoagulant therapy. In addition, if the patient was receiving acetylsalicylic acid (ASA) or antiplatelet agents due to comorbidities, these were added to the ICU treatment.

Laboratory parameters suggestive of thromboembolism were assessed. Minimum and maximum levels of patients' white blood cell (WBC) count, lymphocyte count, platelet count, D-dimer, fibrinogen, ferritin, international normalized ratio (INR), activated partial thromboplastin time (aPTT), prothrombin time (PT) and troponin I levels throughout ICU stay were recorded.

The presence of thromboembolic events was assessed throughout the patient's hospitalization. It was recorded whether the diagnosis was made on the basis of clinical findings and imaging techniques. In cases of clinical suspicion, venous Doppler imaging was performed for deep vein thrombosis (DVT), CT angiography for pulmonary embolism, coronary angiography for MI, cranial CT or diffusion MRI for cerebrovascular event, and arterial Doppler for arterial occlusion, and diagnoses were made in consultation with the consultant physicians of the relevant clinics. Pulmonary embolism (PE) and deep vein thrombosis (DVT) were classified as venous thromboembolism. Occlusion of a central venous catheter, renal replacement therapy, or extracorporeal membrane oxygenation (ECMO) set was also considered venous thromboembolism. Myocardial infarction, cerebrovascular event and peripheral artery occlusion were classified as arterial thromboembolism.

Statistical analysis

Data were analyzed using IBM SPSS Statistics for Windows, Version 22.0 (Released 2013; IBM Corp., Armonk, New York, United States). Number and percentage were used for categorical variables; minimum, maximum, mean, standard deviation, and median tests were used for quantitative data. The Kolmogorov-Smirnov test was performed to analyze the normal distribution of the data and it was found that the data did not have a normal distribution. Therefore, non-parametric tests were used to analyze the data. The chi-square test was used to compare categorical variables. Again, the input values of some laboratory findings for the presence of thromboembolism were analyzed using the ROC curve test and cut-off values were found. P<0.05 was considered significant in the analysis.

## Results

Demographic and ICU admission data

**Figure 1:**
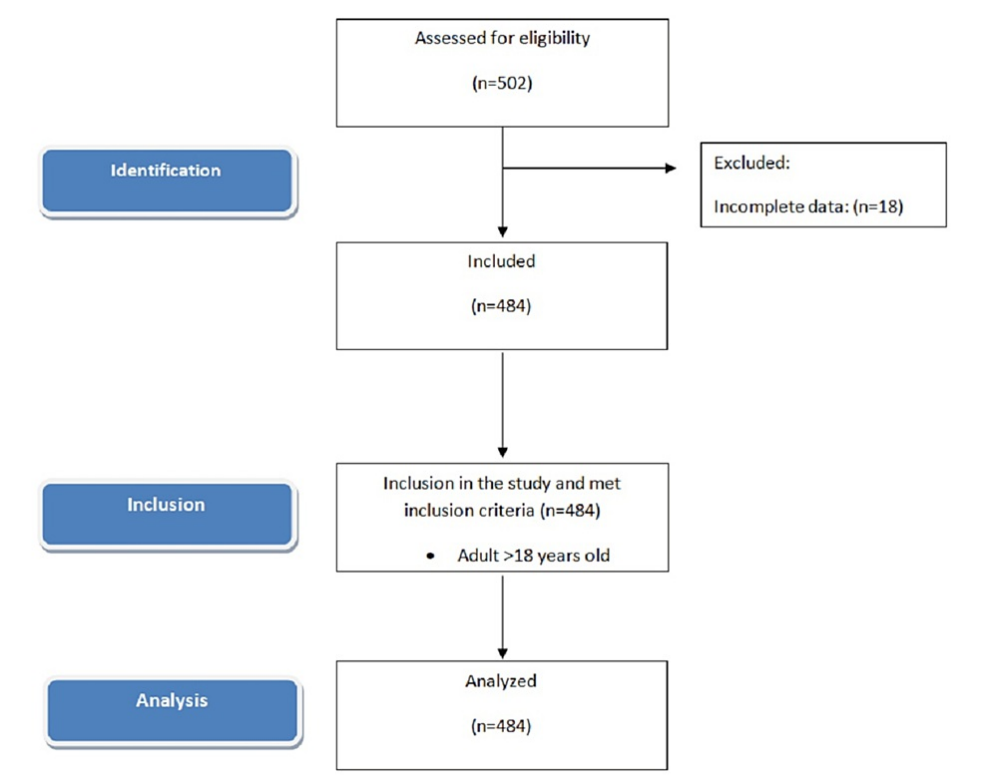
Cohort development flow chart

Data were extracted from 502 patients. All patients were over 18 years of age. Eighteen patients with incomplete data whose laboratory data could not be accessed were excluded from the study (Figure 1). Statistical analysis was performed on data from 484 patients aged 18-98 years (65.87±16.28 years). Of the patients, 248 (51.2%) were female and 236 (48.8%) were male.

When evaluating comorbidities, cardiovascular disease was the most common comorbidity at 51% (n=277), followed by DM at 31%, hypertension at 27%, and malignancies at 19%(Table 1).

**Table 1:** Demographic data of the patients CVS: cardiovascular system; DM: diabetes mellitus

		Number (%) or Mean (SD)
Age (years), mean (SD)		65.8 (16.33)
		N %
Gender	Male	236 (48.8%)
	Female	248 (51.2%)
Comorbidities	CVS Diseases	277 (57.2%)
	DM	165 (31%)
	Hypertension	112 (27.1%)
	Metabolic Diseases	43 (10.4%)
	Neurologic Diseases	48 (11.6%)
	Respiratory System Diseases	49 (11.9%)
	Malignancies	81 (19.6%)
	Renal diseases	49 (11.9%)
	Pregnancy	22 (5.3%)
	Endocrinologic and rheumatologic diseases	9 (2.2%)

The length of stay in the ICU ranged from 1-191 days (12.54±16.48) and the mean acute physiology and chronic health evaluation (APACHE) II score on admission was 18.53±9.85. It was found that 41.5% of the patients had positive polymerase chain reaction (PCR) tests on admission to the ICU and the rate of positive patients reached 69.8% with PCR results obtained during hospitalization. The rates of COVID-19 subtypes, ancestral variant, delta variant, alpha variant, and others were 78.4%, 16%, 4.4%, and 1.2%, respectively, in the positive patients.

General thromboembolism occurred in 14.8%(n=72), venous thromboembolism in 3.5% (n=17), and arterial thromboembolism in 11.3% (n=55) of patients. No statistically significant association was found between PCR results and thromboembolism when the COVID-19 subtypes were assessed; 11.3% (n=30) of the ancestral variants and 12.9% (n=7) of the delta variants developed thromboembolism (Table 2).

**Table 2:** Association of PCR results and COVID-19 subtypes with thromboembolism PCR: polymerase chain reaction; COVID-19: coronavirus disease 2019

		Thromboembolism (-)	Thromboembolism (+)	p-value
PCR results	Negative	121 (82.9%)	25 (17.1%)	0.075
	Positive	301 (89.1%)	37 (10.9%)	
COVID-19 subtype	Ancestral variant	235 (88.7%)	30 (11.3%)	0.458
	Delta variant	47 (87.1%)	7 (12.9%)	
	Alfa variant	15 (100%)	-	
	Other variants	4 (100%)	-	

When patients' comorbidities were compared with the presence of total thromboembolism, a statistically significant difference was found for CVS disease (p<0.01) (Table 3).

**Table 3:** Comorbidities and thromboembolism in patients Chi-square test: values are given as frequency (percentage); p<0.05 statistically significant difference between the groups CVD: cardiovascular disease; DM: diabetes mellitus

Comorbidities	Thromboembolism (-), n (%)	Thromboembolism (+), n (%)	p-value
DM	138 (32.7%)	27 (43.5%)	0.063
CVD	228 (54%)	49 (79%)	<0.001
Hypertension	92 (82.2%)	20 (17.8%)	0.514
Metabolic Diseases	38 (88.4%)	5 (11.6%)	
Neurologic Diseases	38 (79.2%)	10 (20.8%)	
Respiratory System Diseases	41 (11.6%)	8 (13.3%)	
Malignancies	73 (90.2%)	8 (9.8%)	
Renal Diseases	42 (85.8%)	7 (14.2%)	
Pregnancy	21 (95.5%)	1 (4.5%)	
Endocrinologic and Rheumatologic Diseases	8 (88.9%)	1 (11.1%)	

When comparing treatment variables with the presence of thromboembolism, no statistically significant difference was found in the association of different doses of LMWH and no treatment with thromboembolism, whereas a statistically significant difference was found for ASA (p<0.001) and antiplatelet variables (p<0.01) (Table 4).

**Table 4:** Association of treatment variables with thromboembolic events Prophylactic dose: For BMI<40 kg/m^2^, enoxaparin sodium 40 mg once a day subcutaneous; For BMI>40 kg/m^2, ^enoxaparin sodium 40 mg twice a day subcutaneous Treatment dose: 25-30 % higher than the prophylactic dose LMWH: low-molecular-weight heparin; ASA: acetylsalicylic acid

Antithrombotic Agent	Thromboembolism (-), n (%)	Thromboembolism (+), n (%)	p-value
LMWH			
No treatment	21 (87.5%)	3 (12.5%)	0.218
Prophylactic dosage	103 (92%)	9 (8.0%)	
Treatment dosage	298 (83.7%)	50 (14.3%)	
Heparin	5 (62.5%)	3 (37.5%)	0.07
ASA	55 (63.3)	32 (36.7%)	<0.001
Antiplatelet	31 (48.5)	33 (51.5%)	<0.001

When the relationship between some variables of the patients and the presence of thromboembolism was analyzed, age, APACHE II score, D-dimer, fibrinogen, ferritin, PT, INR, troponin values on admission to the intensive care unit were found statistically significant (p<0.005) (Table 5).

**Table 5:** Association of age and laboratory values with thromboembolism APACHE: acute physiology and chronic health evaluation; aPTT: activated partial thromboplastin time; PT: prothrombin time; INR: international normalised ratio; DIC: disseminated intravascular coagulation

	Thromboembolism (-), mean±SD	Thromboembolism (+), mean±SD	p-value
Age (years)	64.96±16.57	71.93±13.2	0.003
Length of Stay in ICU (days)	12.6±16.36	12.17±17.38	0.614
APACHE II Score	18.01±9.67	22±10.44	0.003
D-Dimer (μg/mL)	5.5±11.48	13.05±21.64	0.015
Fibrinogen (mg)	467.54±155.82	427.85±125.55	0.032
Ferritin (ml/ng),	991.55±1096.46	736.51±679.23	0.015
Platelet count (10^3^/μL)	242405.21±118151.51	220741.93±96683.01	0.096
aPTT (second)	28.81±23.91	29.85±14.01	0.118
PT (second)	15.04±10.95	15.14±5.46	0.015
INR	1.3±1.08	1.31±0.52	0.012
WBC (10^3^/μL)	12504.74±12504.48	11674.2±7114.81	0.986
Lenfosit (10^3^/μL)	2139.1±10395.88	1298.38±2565.37	0.112
Troponin (ng/mL)	365.8±2210.5	1385.06±5335.81	0.012
DIC score	2.75±2.31	3.03±2.26	0.307

Receiver operating characteristic curve (ROC) analysis was performed according to the presence of general thromboembolism in the patients' admission laboratory results. Cut-off values for predicting the development of thromboembolism in intensive care patients diagnosed with COVID-19 were 2.565 for D-dimer, 435.51 for fibrinogen, 633.55 for ferritin, 1.155 for INR, and 0.085 for troponin I (Table 6).

**Table 6:** ROC analysis of various laboratory parameters associated with thromboembolism INR: international normalized ratio; ROC: receiver operating characteristic curve; AUC: area under the curve

	AUC	Cut-off value	p-value	Sensitivity (%)	Specificity (%)	Confidence interval (95%), min.-max.
D-Dimer (μg/mL)	0.596	2.565	0.015	57.4	42.9	0.511-0.681
Fibrinogen (mg)	0.413	435.51	0.032	42.4	56.8	0.343-0.484
Ferritin (ml/ng)	0.403	633.55	0.015	43.8	57.5	0.325-0.481
INR	0.599	1.155	0.012	59.7	40.1	0.523-0.674
Troponin I (ng/mL)	0.605	0.085	0.013	57.4	40.8	0.52-0.69

## Discussion

This retrospective study showed that among the patients diagnosed with COVID-19 in the ICUs of our hospital, the general thromboembolism rate was 14.8%, the arterial thromboembolism rate was 11.3%, and the venous thromboembolism rate was 3.5%. It was found that different doses of LMWH were not effective in preventing thromboembolism. However, ASA and other antiplatelet agents have been shown to reduce the development of thromboembolism. The presence of cardiovascular disease, age, high levels of APACHE II score, D-dimer, ferritin, fibrinogen, and troponin I, and low levels of PT and INR at admission to the ICU have been shown to be risk factors for the development of thromboembolism. When the effect of laboratory values on the development of thromboembolism was evaluated, cut-off values were found to be 2.565 for D-dimer, 435.51 for fibrinogen, 633.55 for ferritin, 1.155 for INR, and 0.085 for troponin I.

Many studies have reported that COVID-19 infection leads to thromboembolic complications [7]. In these studies, the incidence of thromboembolism has also been found to be in a wide range. In published studies, the incidence of thromboembolism has been reported to be as high as 13-60% [11], and in a post-mortem study by Dolhnikof et al., the incidence was reported to be 80% [12]. In the present study, the overall incidence of thromboembolism was 14.8%, while the incidence of arterial and venous thromboembolism was 11.6% and 3.5%, respectively. In one of the first studies on this subject, Cui et al. investigated the incidence of VTE in patients with severe COVID-19 pneumonia, all patients were scanned with venous Doppler, and the incidence of VTE was found to be 25% [8]. In the studies by Lodgiani et al. [13] and Klok et al. [14], the incidence was reported to be 27% and 31%, respectively. These results are higher than ours. In the multicentre study by Helms et al., the incidence of thromboembolism in patients admitted to intensive care was 16% [15]. Similarly, the study by Middledorp et al. reported a VTE incidence of 16% [16], which is similar to our results.

When we analyzed the incidence of arterial and venous thromboembolism separately, the incidence of venous thromboembolism in our study was 3.5%, which was lower than that in the literature. We categorized cases of arterial thromboembolism as cerebrovascular events, myocardial infarction, and peripheral arterial embolism. The frequency of clinically suspected cerebrovascular events in our patients was 2.9% (n=14), while the number of confirmed cases was 13 patients with a frequency of 2.7%. The frequency of confirmed myocardial infarction was 2.5% and the frequency of confirmed peripheral arterial thrombosis was 2.1%. These results were similar to those found by Lodgiani et al. [13] Similarly, in the study by Klok et al., the prevalence of arterial thrombosis in patients with cerebrovascular stroke was reported to be 23.7% [14].

We hypothesized that the low incidence of venous thromboembolism in our study is due to the fact that advanced investigations such as venous Doppler or pulmonary CT angiography were performed in the ICUs of our hospital only in the presence of clinical suspicion. On the other hand, there are studies that report daily screening of patients for DVT with Doppler ultrasound [8].

The PCR results of 338 patients were positive in the current study and when we analyzed the results, the frequencies of the first variant, which we call the ancestral variant, the delta variant, the alpha variant, and the Brazilian-South African variant strains (grouped under the title of other variants) were determined to be 78.4%, 16%, 4.4%, and 1.2%, respectively. Looking at the literature, the number of studies investigating the association between variants and thromboembolic complications is very small and the results obtained are weak [17-19]. It has been reported in the literature that the delta variant is more infectious due to its increased affinity for the angiotensin-converting enzyme 2 (ACE2) receptor on the endothelium, leading to increased fibrinolysis and platelet aggregation. The natural consequence is an increase in the incidence of thromboembolic events [20]. In a study comparing the delta and Omicron variants, it was found that the Omicron variant, known to have a milder course, was more lethal in intensive care patients than the delta variant, known to have a more aggressive course, and it was concluded that the protective effect of vaccination against the delta variant was effective in this outcome, but the association with thromboembolism was not mentioned [18]. In the present study, the effect of variants on thromboembolic complications was not statistically significant. However, similar to the literature, the frequency of thromboembolism was higher in patients with the delta variant compared to the ancestral variant (12.9% > 11.3%).

In the ICUs of our hospital, antithrombotic treatment was administered according to the national COVID-19 guidelines for diagnosis and treatment. According to this guideline, the treatment is graded according to the severity of the disease and the presence of coagulopathy, but the first choice drug is recommended as LMWH or heparin [21]. In addition, if the patient was on ASA or other antiplatelet agents due to comorbidities, treatment was continued in the intensive care unit. When the data of the patients included in our study were analyzed, it was found that 95% of the patients used LMWH, 18% used ASA, 13% used other antiplatelet agents, and 1.7% used heparin. When the effect of the drugs used by the patients on thromboembolic outcome was analyzed, the effect of LMWH and heparin was not found to be statistically significant, whereas the use of ASA and antiplatelet agents was found to be statistically significant (<0.001). In a randomized controlled trial (RCT) conducted by Spyropoulos et al., LMWH at a therapeutic dose was found to reduce the risk of thromboembolism and death compared with standard heparin in patients with high D-dimer levels, but the same effect was not shown in ICU patients [22]. On the other hand, Tang et al. showed that LMWH was associated with a better prognosis in patients with high D-dimer levels [23]. Information on the use of ASA and other antiplatelet agents remains limited

CVS diseases, which included coronary heart disease, congestive heart failure, and arrhythmias, were the most prevalent comorbidities among the patients in our study, accounting for 57.2% of all cases. Diabetes mellitus came in second place (31%) and hypertension came in third place (27.1%). This was followed by malignancy at 19.6%, and it was seen that 50.2% of patients had more than one comorbidity. When we analyzed the relationship between comorbidity and thromboembolism, the presence of cardiovascular disease and advanced age were significant for thromboembolism. It has been reported in the literature that advanced age, hypertension, diabetes mellitus, hyperlipidemia, and multiple comorbidities are associated with a high risk of thromboembolism [16,18,24-26].

COVID-19 has been reported to cause changes in many laboratory values in patients. In the literature, it has been reported to cause abnormalities in many cellular and biochemical parameters such as leukocytosis, leukopenia, neutrophilia, hypoalbuminemia, ferritin, troponin, and myoglobin [7]. In our study, D-dimer, ferritin, fibrinogen, WBC, platelet count, troponin, PT, aPTT, INR, and lymphocyte levels were monitored. D-dimer (95%CI 0.511-0.681), fibrinogen (95%CI 0.343-0.484), ferritin (95%CI 0.325-0.481), INR (95%CI 0.523-0.674), and troponin (95%CI 0.52-0.69) were statistically significant for the development of thromboembolism. When coagulopathy develops in the body, the first signs of thrombosis appear as increased fibrin degradation products, especially increased D-dimer levels, and in the late period, prolonged PT and aPTT, increased platelet count and fibrinogen levels are already known from the literature [27]. Therefore, it is normal that the D-dimer level was found to be significant in our study. Ferritin stood out as an indicator of ARDS development in COVID-19 patients. Ferritin levels are increased by inflammatory mediators such as tumor necrosis factor (TNF), interleukin (IL)-1, IL-6, and IL-18 in the body. Increased ferritin is an indicator of macrophage activation. The underlying cause of thrombosis in COVID-19 is due to an increased immune response, which can be referred to as immunothrombosis. In this case, ferritin is an indicator of immunothrombosis [28,29]. Similar to our study, D-dimer and ferritin were reported as independent risk factors for thrombosis in the study by Oba et al. [26]. Similar to our study, Ashraf et al. reported that troponin I may have a positive predictive value for thrombosis in COVID-19 patients [30].

In the ROC analysis of laboratory values, cut-off values were 2.565 μg/L for D-dimer, 435 for fibrinogen, 633 for ferritin, and 0.085 for troponin I. Studies have shown that D-dimer levels are elevated in 36% of COVID-19 patients and that D-dimer levels vary according to disease severity. While the mean D-dimer value was 0.5mg/L in mild cases, the mean value was 2.5mg/L in severe cases [23]. In the study by Oba et al., the cut-off value for ferritin level was reported to be 433ng/mL [26]. Fibrinogen is one of the important members of thrombosis as it is involved in the last step of the coagulation cascade and appears as an acute phase reactant and one of the scoring parameters in the diagnosis of DIC [7]. In this respect, its role in the prediction of thromboembolism is clear. The troponin I level has also been found in studies to be a marker of poor prognosis together with D-dimer [30].

The first reports about the thromboembolic complication of COVID-19 were made in China, the origin of the disease, and immediately afterward in Italy [8,13,14,16]. After the first reports mentioned, articles have been published from different countries reporting the incidence of thromboembolism in their populations [26]. Our study is the first study on the incidence of thromboembolism in intensive care patients in Turkey.

Study limitations

The current study had its share of limitations. An important limitation of our study is the retrospective design of the study. Second, because the study included only patients in intensive care, the results cannot be generalized to the whole patient population. Criteria for ICU admission may have been slightly different during the pandemic period. After the study period, the Omicron variant became the dominant variant in Türkiye and the number of intensive care admissions decreased. On the other hand, the representation rate of the alpha variant and other variants is low in our study population. As such, the results of the study may not give complete information about alpha and other variants. As our study was a single-center study, its validity for the general Turkish population is limited.

## Conclusions

This study examines the risk of arterial and venous thromboembolism as well as the general thromboembolism risk in patients diagnosed with COVID-19 hospitalized in ICUs in Türkiye. Our study demonstrates the need for effective anticoagulant treatment to prevent thromboembolism. Although LMWH is the first choice, it may be appropriate to add ASA and other antiplatelet agents in patients without bleeding risk and with severe coagulopathy. The study shows that more conservative anticoagulant therapy may reduce the risk of thromboembolism in patients with comorbidities that affect both immunity and vessel wall structure, such as thromboembolic risk, advanced age, cardiovascular disease, and patients with elevated levels of D-dimer, troponin, ferritin, and fibrinogen. To identify high-risk populations and establish suitable interventions for thrombotic complications in COVID-19, more research would be needed.
